# A cascaded clinical-ultrasound-biochemical model for precise prediction before thyroid nodule fine-needle aspiration biopsy

**DOI:** 10.3389/fmed.2025.1641266

**Published:** 2025-09-18

**Authors:** Shuhang Gao, Bojia Liu, Mengying Tong, Yalin Zhu, Lina Wang, Linyao Du, Chang Shi, Mei Han, Ying Che

**Affiliations:** ^1^Department of Ultrasound, The First Affiliated Hospital of Dalian Medical University, Dalian, China; ^2^College of Humanities and Social Sciences, Dalian Medical University, Dalian, China; ^3^Department of Pathology, The First Affiliated Hospital of Dalian Medical University, Dalian, China

**Keywords:** fine-needle aspiration, logistic regression, ultrasound imaging, thyroid nodules, precision medicine

## Abstract

**Objectives:**

Determining the nature of thyroid nodules through a single fine-needle aspiration (FNA) biopsy is not feasible for approximately one-third of patients. We developed a predictive model to assist FNA decision-making and reduce unnecessary FNAs.

**Methods:**

This retrospective study consecutively included patients who underwent ultrasound-guided FNA between March 2018 and March 2023. Patients were divided into a training dataset (70%) and a validation dataset (30%). Univariate analysis was performed within the training dataset using Kruskal–Wallis test for continuous variables and chi-square test or Fisher’s exact test for categorical variables. Variables with significance were entered into multivariate logistic regression. The prediction model (B-Model) was constructed using a cascaded three-stage logistic regression framework: Stage I distinguished benign from non-benign nodules, Stage II differentiated malignant from non-malignant nodules, Stage III separated follicular neoplasm from indeterminate/atypia nodules. Model performance was assessed in the validation dataset using sensitivity (SEN), specificity (SPE), and accuracy (ACC). The reduction in repeat FNA facilitated by the B-Model was calculated.

**Results:**

Training and validation datasets included 1,573 and 672 cases, respectively. The overall SEN, SPE and ACC of the B-Model were 84.7%, 76.7% and 60.1% in the validation dataset. The application of the B-Model reduced the number of patients requiring repeat FNA from 255 to 153, resulting in a 40.0% reduction.

**Conclusion:**

The B-Model demonstrated robust predictive performance, facilitating the optimization of pre-FNA diagnostic workflows, significantly reducing unnecessary repeat FNAs, and advancing precision in thyroid nodule management.

## 1 Introduction

Thyroid nodules (TNs) are common in the general population, with a global incidence ranging from 19 to 68%. Most nodules are benign, with 7–15% being malignant (1–3). Given the differences in pathogenesis, biologic behavior, and clinical manifestations, there are significant variations in treatment and prognosis among different pathologic types and subtypes of TNs (4). In recent years, the advent and dissemination of treatment technologies, such as ablation, targeted therapy, immunotherapy, and traditional Chinese medicine, have revolutionized the management of TNs (5). To provide patients with more precise and personalized treatment strategies, accurate pathologic diagnosis of TNs is crucial.

Ultrasound (US)-guided fine-needle aspiration biopsy (FNA) is a safe and effective method for obtaining thyroid cells and is currently the preferred approach for diagnosing TNs (1, 6–8). The Bethesda System for Reporting Thyroid Cytopathology (BSRTC), which is widely adopted globally, aims to unify the terminology used in pathology reports and achieve standardized reporting (9–11). BSRTC II, V, and VI are distinctly labeled as benign, suspicious for malignancy, and malignant. Conversely, BSRTC I, III, and IV encompass nondiagnostic, atypia of undetermined significance, and follicular neoplasm, respectively, which lack definitive diagnoses and exhibit a potential occurrence range of 20–34% (10–13). Multiple guidelines suggest that comprehensive management should be performed based on clinical risk factors in accordance with the patient’s wishes. Repeat FNA (rFNA) is highly recommended for BSRTC I nodules. For BSRTC III, a range of options are advised, including rFNA, rFNA with molecular testing, diagnostic lobectomy, and surveillance. Concerning BSRTC IV, the recommended approach encompasses rRNA coupled with molecular testing or diagnostic lobectomy (1, 6, 14). Therefore, approximately one-third of patients may require two FNA procedures to achieve a more precise diagnosis. Even after undergoing two FNAs, some patients still confront diagnostic ambiguity, which ultimately requires thyroidectomy. This undoubtedly increases patient exposure to invasive procedures, prolongs waiting time, and imposes a significant financial burden.

This study aimed to devise a predictive model (B-Model) for BSRTC categorization of FNA that identifies nodules that cannot be determined solely through FNA so that we can minimize ineffective punctures, maximize the diagnostic efficiency of FNA, and ultimately promote precision medicine.

## 2 Materials and methods

### 2.1 Patients

**Figure 1:**
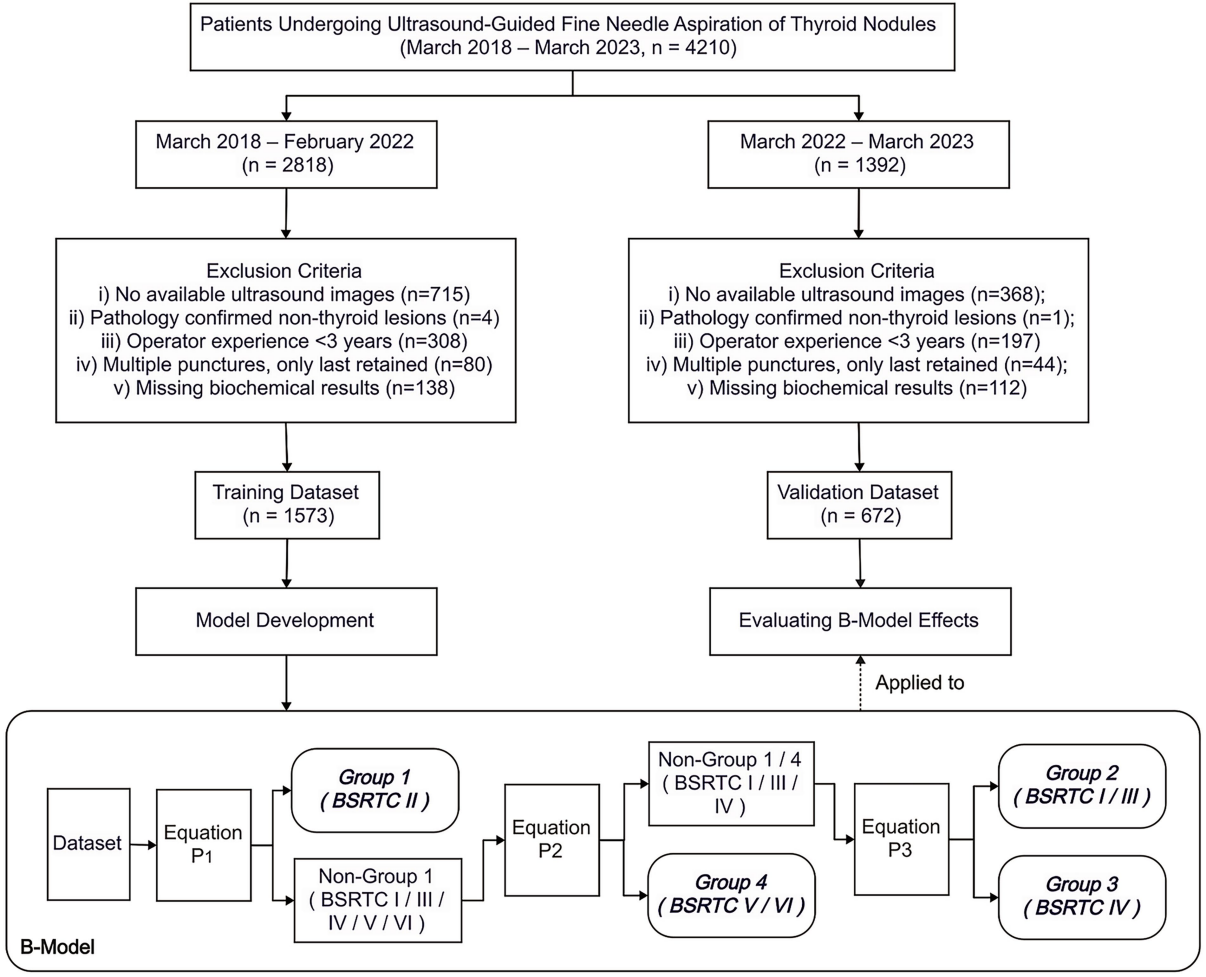
Study flow diagram of patient enrollment, dataset allocation, and B-Model development. Study flow diagram showing inclusion and exclusion criteria, patient enrollment, and dataset allocation into training and validation cohorts, with datasets divided chronologically (March 2018–February 2022 for training, March 2022–March 2023 for validation). Architecture of the cascaded logistic regression model (B-Model), in which three logistic regression equations were sequentially linked: Equation P_1_ distinguished benign from non-benign nodules (Group 1 vs. non-Group 1); Equation P_2_ differentiated malignant from non-malignant nodules (Group 4 vs. non-Group 4); and Equation P_3_ further separated follicular neoplasm from indeterminate/atypia nodules (Group 3 vs. Group 2). BSRTC, Bethesda System for Reporting Thyroid Cytopathology [Flowchart design: Boardmix Online Platform (https://boardmix.cn)].

This single-center retrospective study consecutively included patients who underwent US-FNA of TNs between March 2018 and March 2023 (*n* = 4,210). To evaluate temporal generalizability, the dataset was divided chronologically into two cohorts: March 2018 to February 2022 (training dataset) and March 2022 to March 2023 (validation dataset). Exclusions criteria included: absence of ultrasound images, pathology-confirmed non-thyroid lesions, operator experience <3 years, multiple punctures (only the last result retained), and missing biochemical data. After exclusions, the final study population consisted of 1,573 patients in the training dataset and 672 patients in the validation dataset, with an approximate ratio of 7:3 between the two cohorts. The overall study design and patient selection flow are illustrated in Figure 1.

### 2.2 Acquisition of clinical information and biochemical results

Clinical information and biochemical results for all patients were obtained from an electronic medical data management system. The following clinical features were recorded: patient’s age and sex. Biochemical results included free triiodothyronine (FT3), free thyroxine (FT4), thyroid-stimulating hormone (TSH), antithyroid peroxidase autoantibody (A-TPO), thyroglobulin antibody (A-TG), thyroglobulin (TG), and thyrotropin receptor antibody (TRAb). All biochemical tests were conducted within 1 month of the FNA.

### 2.3 Cytopathology acquisition and grouping

All cytopathologic examinations were performed by two pathologists with >8 years of thyroid cytopathology experience and subsequently reviewed by a senior pathologist with >15 years of experience. Findings were classified according to the 2023 revision of BSRTC into four groups: Group 1 (BSRTC II), Group 2 (BSRTC I/III), Group 3 (BSRTC IV), and Group 4 (BSRTC V/VI).

### 2.4 Ultrasound image acquisition and interpretation

Ultrasound data were retrieved from the institutional imaging system. Two US radiologists (>7 years of thyroid imaging experience) independently assessed thyroid echotexture, nodule position, capsule distance, size, volume, composition, echogenicity, echotexture, margin, shape, orientation, calcifications, posterior features, halo and Adler’s semiquantitative grading for nodule blood flow (Grades 0–3). Discrepancies were resolved by consensus with a senior radiologist (>20 years of experience).

### 2.5 Statistical analysis

SPSS statistical software (version 20.0; IBM Corporation, Armonk, NY, USA) was used for the statistical analysis. Baseline characteristics between the training and validation datasets were compared using the Mann–Whitney U test for continuous variables and the chi-square or Fisher’s exact test for categorical variables. Univariate analyses were further performed within the training dataset to identify factors associated with pathological classification, applying the Kruskal–Wallis test for continuous variables and the chi-square or Fisher’s exact test for categorical variables across the four groups. A *p*-value of <0.05 was considered statistically significant.

The prediction model (B-Model) was developed using multivariable logistic regression in SPSS based on training dataset, and it adopted a three-stage architecture as illustrated in Figure 1: (1) distinguished benign from non-benign nodules (Group 1 vs. non-Group 1) by Equation P_1_; (2) differentiated malignant from non-malignant nodules (Group 4 vs. non-Group 4) by Equation P_2_; (3) separated follicular neoplasm from indeterminate/atypia nodules (Group 3 vs. Group 2) by Equation P_3_. Each equation had two versions: one that included biochemical indicators as independent variables P(w), and another that did not include biochemical indicators as independent variables P(w/o). For other special circumstances, a supplementary version was designed P(c). Multivariable logistic regression analyses with backward stepwise selection were applied to identify independent variables *x_1-i_*. Based on clinical significance or published reports, we graded each risk factor, selected an appropriate grade as the baseline risk reference value, and recorded the score as 0 (1, 6, 13). *β_0-i_* is the regression coefficient of each independent variable. Using these parameters, we calculated P as the dependent variable corresponding to each risk factor classification using the following formula, where *exp* denotes the natural exponential function:


y=β0+β1x1+β2x2+…+βixi

$$P=\frac{\exp^{y}}{1+\exp^{y}}=\frac{\exp^{\beta 0+\beta 1x1+\beta 2x2+\ldots +\text{βixi}}}{1+\exp^{\beta 0+\beta 1x1+\beta 2x2+\ldots +\text{βixi}}}$$

The dependent variable *P* in the equation above uses 0.5 as a threshold value. Similar cascaded/sequential logistic regression approaches have been applied in recent medical prediction studies to improve classification performance and manage class imbalance (15–17).

The data in the validation dataset were used to select the equations and validate the performance of the prediction models. By substituting the data into previously established equations and considering the actual pathologic results as the gold standard, the sensitivity (SEN), specificity (SPE), accuracy (ACC), positive predictive rate (PPV), negative predictive rate (NPV) and area under the receiver operating characteristic curve (AUC-ROC) of each equation were evaluated. Finally, the rate of reduction in rFNAs after the B-Model implementation was calculated using the following equation:


$$\text{rFNA reduction rate}\;(\%)=\frac{\text{True Group}\;2/3-B-\text{Model}\;\mathrm{FN}\;}{\text{True Group}\;2/3}\times 100\%$$

(FN: True Group 2/3 cases incorrectly classified as Group 1/4 by B-Model).

## 3 Results

### 3.1 Patient characteristics

In the training dataset, the final cohort included 1,573 patients [median age: 48 years (IQR: 38–57)] of the initial 2,818 patients, after the exclusion of 1,245 patients. In the validation dataset, the final cohort included 672 patients [median age: 50 years (IQR: 40–58)] of the initial 1,392 patients, after excluding 720 patients. The patient characteristics, US features, and biochemical results are shown in Table 1. Overall, no significant statistical differences were observed between two cohorts for most baseline characteristics except three laboratory indicators (FT4, A-TG, and A-TPO; *p* = 0.047, <0.001, and 0.002, respectively). These differences likely reflect case-mix shifts from time-based cohort division and variability in laboratory assays.

**Table 1:** Comparison of baseline clinical characteristics and ultrasound features of thyroid nodules between the training and validation datasets ^a,b^.

Characteristics	Training dataset (*n* = 1,573)	Validation dataset (*n* = 672)	*p*-value
Age (y)	48 (38, 57)	50 (40, 58)	0.079
Sex			0.117
Female	1,252 (79.6)	515 (76.6)	
Male	321 (20.4)	157 (23.4)	
Thyroid echotexture			0.290
Homogeneous	1,211 (77.0)	531 (79.0)	
Heterogeneous	362 (23.0)	141 (21.0)	
Lobe			0.076
Right	837 (53.2)	324 (48.2)	
Left	633 (42.0)	294 (43.8)	
Isthmus	103 (6.5)	54 (8.0)	
Position			0.184
Superior	330 (21.0)	132 (19.6)	
Middle	712 (45.3)	286 (42.6)	
Inferior	531 (33.8)	254 (37.8)	
Capsule distance (mm)			0.114
>2	463 (29.4)	175 (26.0)	
≤2	1,110 (70.6)	497 (74.0)	
Size (mm)			0.072
≤5.0	354 (22.5)	129 (19.2)	
5.1–10.0	553 (35.2)	219 (32.6)	
10.1–40.0	578 (36.7)	282 (42.0)	
>40.0	88 (5.6)	42 (6.3)	
Volume (mL)	0.20 (0.05, 1.56)	0.30 (0.06, 1.89)	
Composition			0.529
Solid	1,304 (82.9)	540 (80.4)	
Predominantly solid	139 (8.8)	69 (10.3)	
Predominantly cystic	55 (3.5)	25 (3.7)	
Spongiform	75 (4.8)	38 (5.7)	
Echogenicity			0.331
Markedly hypoechoic	309 (19.6)	131 (19.5)	
Hypoechoic	897 (57.0)	365 (54.3)	
Isoechoic/ hyperechoic	367 (23.3)	176 (26.2)	
Nodule echotexture			0.157
Homogeneous	872 (55.4)	350 (52.1)	
Heterogeneous	701 (44.6)	322 (47.9)	
Margin			0.427
Smooth	866 (55.1)	357 (53.1)	
Ill-defined	707 (44.9)	315 (46.9)	
Shape			0.880
Oval-to-round	1,126 (71.6)	479 (71.3)	
Lobulated	74 (4.7)	29 (4.3)	
Irregular/extra-thyroidal extension	373 (23.7)	164 (24.4)	
Orientation			0.400
Wider-than-tall	885 (56.3)	391 (58.2)	
Taller-than-wide	688 (43.7)	281 (4.8)	
Calcifications			0.653
Absent	1,136 (72.2)	479 (71.3)	
Macrocalcifications	148 (9.4)	66 (9.8)	
Microcalcifications	248 (15.8)	102 (15.2)	
Peripheral calcifications	19 (1.2)	13 (1.9)	
More than two forms	22 (1.4)	12 (1.8)	
Posterior features			0.731
Absent	1,242 (79.0)	530 (78.9)	
Enhancement	247 (15.7)	101 (15.0)	
Shadowing	84 (5.3)	41 (6.1)	
Halo			0.216
Absent	1,361 (86.5)	590 (87.8)	
Uniform halo	24 (1.5)	15 (2.2)	
Uneven halo	188 (12.0)	67 (9.9)	
Blood flow			0.735
Grade 0	796 (50.6)	355 (52.8)	
Grade 1	385 (24.5)	163 (24.3)	
Grade 2	230 (14.6)	89 (13.2)	
Grade 3	162 (10.3)	64 (9.7)	
TSH (μIU/mL)	1.80 (1.17, 2.66)	1.81 (1.20, 2.70)	0.592
FT3 (pmol/L)	4.43 (4.09, 4.73)	4.32 (4.05, 4.72)	0.129
FT4 (pmol/L)	15.97 (14.64, 17.37)	16.45 (14.93, 17.83)	0.047*
A-TG (IU/mL)	17.29 (13.82, 27.71)	15.17 (11.32 31.73)	0.000**
A-TPO (IU/mL)	12.56 (9.19, 18.00)	15.39 (8.97, 22.71)	0.002**
TG (ng/mL)	24.06 (10.19, 76.87)	22.65 (9.67, 54.69)	0.056
TRAb (IU/L)	1.13 (0.80, 1.44)	1.14 (0.80, 1.57)	0.146

^a^ Continuous variables are presented as medians (Q1, Q3), and categorical variables are presented as numbers and percentages. ^b^ *p*-values were calculated using the Mann–Whitney U test for continuous variables and the chi-square test or Fisher’s exact test for categorical variables. *: *p*-value < 0.05, **: *p*-value < 0.01. Asterisks indicate statistically significant differences. A-TG, thyroglobulin antibody; A-TPO, antithyroid peroxidase autoantibody; FT3, free triiodothyronine; FT4, free thyroxine; TG, thyroglobulin; TRAb, thyrotropin receptor antibody; TSH, thyroid-stimulating hormone.

### 3.2 Factors influencing pathology

In the training dataset, univariate analysis identified significant differences (*p* < 0.05) in 2 patient characteristics, 15 US features, and 4 biochemical markers across the groups (Table 2). Specifically, thyroid echogenicity and A-TG levels were significantly different between Groups 1 and 3 (*p* = 0.047 and *p* = 0.046, respectively) whereas FT4 levels were significantly different between Groups 2 and 4 (*p* = 0.032). All significant variables were included as independent covariates in the subsequent multivariate analysis.

**Table 2:** Patient clinical characteristics and ultrasound findings of the nodules associated with grouping in the training dataset ^a-c^.

Characteristics	Group 1 (*n* = 455)	Group 2 (*n* = 504)	Group 3 (*n* = 76)	Group 4 (*n* = 538)	*p*-value
Age (y)	50 (40, 58)	49 (39, 58)	50 (41, 60)	44 (36, 52)	0.000**
Sex					0.000**
Female	385 (84.6)	410 (81.3)	56 (73.7)	401 (74.5)	
Male	70 (15.4)	94 (18.7)	20 (26.3)	137 (25.5)	
Thyroid echotexture					0.130
Homogeneous	340 (74.7)	379 (75.2)	61 (80.3)	43 (80.1)	
Heterogeneous	115 (25.3)	125 (24.8)	15 (19.7)	10 (19.9)	
Lobe					0.001**
Right	257 (56.5)	257 (51.0)	37 (48.7)	286 (53.2)	
Left	176 (38.7)	224 (44.4)	35 (46.1)	198 (36.8)	
Isthmus	22 (4.8)	23 (4.6)	4 (5.3)	54 (10.0)	
Position					0.000**
Superior	62 (13.6)	115 (22.8)	9 (11.8)	144 (26.8)	
Middle	206 (45.3)	221 (43.8)	32 (42.1)	253 (47.0)	
Inferior	187 (41.1)	168 (33.3)	35 (46.1)	141 (26.2)	
Capsule distance (mm)					0.041*
>2	125 (27.5)	166 (32.9)	14 (18.4)	158 (29.4)	
≤2	330 (72.5)	338 (67.1)	62 (81.6)	380 (70.6)	
Size (mm)					0.000**
≤5.0	35 (7.7)	168 (33.3)	1 (1.3)	150 (27.9)	
5.1–10.0	105 (23.1)	161 (31.9)	17 (22.4)	270 (50.2)	
10.1–40.0	265 (58.2)	149 (29.6)	49 (64.5)	115 (21.4)	
>40.0	50 (11.0)	26 (5.2)	9 (11.8)	3 (0.6)	
Volume (mL)	1.73 (0.18, 6.77)	0.12 (0.30, 0.79)	1.50 (0.323, 4.41)	0.11 (0.04, 0.28)	0.000**
Composition					0.000**
Solid	287 (63.1)	425 (84.3)	65 (85.5)	527 (98.0)	
Predominantly solid	84 (18.5)	38 (7.5)	9 (11.8)	8 (1.5)	
Predominantly cystic	35 (7.7)	17 (3.4)	1 (1.3)	2 (0.4)	
Spongiform	49 (10.8)	24 (4.8)	1 (1.3)	1 (0.2)	
Echogenicity					0.000**
Markedly hypoechoic	33 (7.3)	91 (18.1)	12 (15.8)	173 (32.2)	
Hypoechoic	175 (38.5)	311 (61.7)	52 (68.4)	359 (66.7)	
Isoechoic/hyperechoic	247 (54.3)	102 (20.2)	12 (15.8)	6 (1.1)	
Nodule echotexture					0.000**
Homogeneous	217 (47.7)	310 (61.5)	38 (50.0)	307 (57.1)	
Heterogeneous	238 (52.3)	194 (38.5)	38 (50.0)	231 (42.9)	
Margin					0.000**
Smooth	332 (73.0)	252 (50.0)	63 (82.9)	219 (40.7)	
Ill-defined	123 (27.0)	252 (50.0)	13 (17.1)	319 (59.3)	
Shape					0.000**
Oval-to-round	385 (84.6)	376 (74.6)	62 (81.6)	303 (56.3)	
Lobulated	24 (5.3)	17 (3.4)	7 (9.2)	26 (4.8)	
Irregular/extra-thyroidal extension	46 (10.1)	111 (22.0)	7 (9.2)	209 (38.8)	
Orientation					0.000**
Wider-than-tall	371 (81.5)	287 (56.9)	62 (81.6)	165 (30.7)	
Taller-than-wide	84 (18.5)	217 (43.1)	14 (18.4)	373 (69.3)	
Calcifications					0.000**
Absent	384 (84.4)	369 (73.2)	55 (72.4)	328 (61.0)	
Macrocalcifications	35 (7.7)	59 (11.7)	10 (13.2)	44 (8.2)	
Microcalcifications	31 (6.8)	62 (12.3)	9 (11.8)	146 (27.1)	
Peripheral calcifications	5 (1.1)	10 (2.0)	2 (2.6)	2 (0.4)	
More than two forms	0 (0.0)	4 (0.8)	0 (0.0)	18 (3.3)	
Posterior features					0.000**
Absent	339 (74.5)	389 (77.2)	36 (47.4)	478 (88.8)	
Enhancement	107 (23.5)	76 (15.1)	38 (50.0)	26 (4.8)	
Shadowing	9 (2.0)	39 (7.7)	2 (2.6)	34 (6.3)	
Halo					0.000**
Absent	354 (77.8)	445 (88.3)	51 (67.1)	511 (95.0)	
Uniform halo	7 (1.5)	5 (1.0)	2 (2.6)	10 (1.9)	
Uneven halo	94 (20.7)	54 (10.7)	23 (30.3)	17 (3.2)	
Blood flow					0.000**
Grade 0	161 (35.4)	298 (59.1)	5 (6.6)	332 (61.7)	
Grade 1	136 (29.9)	98 (19.4)	14 (18.4)	137 (25.5)	
Grade 2	94 (20.7)	59 (11.7)	25 (32.9)	52 (9.7)	
Grade 3	64 (14.1)	49 (9.7)	32 (42.1)	17 (3.2)	
TSH (μIU/mL)	1.65 (1.00, 2.62)	1.89 (1.25, 2.95)	1.93 (1.39, 2.34)	1.77 (1.25, 2.46)	0.044*
FT3 (pmol/L)	2.26 (4.12, 4.74)	4.34 (4.06, 4.66)	4.76 (4.45, 5.37)	4.37 (4.09, 4.73)	0.035*
FT4 (pmol/L)	15.98 (14.51, 17.46)	15.74 (14.56, 16.98)	16.12 (14.34, 17.17)	16.09 (14.74, 17.48)	0.217
A-TG (IU/mL)	18.08 (14.44, 28.99)	17.31 (12.94, 39.23)	15.00 (15.00, 112.55)	17.12 (13.78, 22.44)	0.079
A-TPO (IU/mL)	12.47 (9.10, 16.67)	11.97 (8.34, 16.65)	28.00 (14.46, 38.66)	12.72 (9.47, 18.65)	0.000**
TG (ng/mL)	46.63 (18.28, 137.28)	24.87 (10.88, 101.10)	35.78 (12.97, 204.05)	17.15 (7.57, 37.48)	0.000**
TRAb (IU/L)	1.13 (0.83, 1.40)	1.11 (0.80, 1.44)	0.44 (0.30, 0.93)	1.15 (0.80, 1.48)	0.000**

^a^ Continuous variables are presented as medians (Q1, Q3), and categorical variables are presented as numbers and percentages. ^b^ *p*-values were calculated using the Kruskal–Wallis test for continuous variables and chi-square test or Fisher’s exact test for categorical variables. ^c^ If the variable has a theoretical value of <10, it can be obtained using Fisher’s exact test. *: *p*-value < 0.05, **: *p*-value < 0.01. Asterisks indicate statistically significant differences. A-TG, thyroglobulin antibody; A-TPO, antithyroid peroxidase autoantibody; FT3, free triiodothyronine; FT4, free thyroxine; TG, thyroglobulin; TRAb, thyrotropin receptor antibody; TSH, thyroid-stimulating hormone.

### 3.3 Construction of equations P_1_, P_2_, and P_3_

There versions of Equation P_1_ were derived: P_1_(w/o) (*χ*^2^ = 457.323, *p* < 0.001), P_1_(w) (*χ*^2^ = 300.627, *p* < 0.001), and P_1_(c) (*χ*^2^ = 300.627, *p* < 0.001). P_1_(c) was generated by cross-validation to address the absence of biochemical indicators in P_1_(w). Two versions of Equation P_2_ were developed: P_2_ (w/o) (*χ*^2^ = 324.479, *p* < 0.001) and P_2_ (w) (*χ*^2^ = 198.300, *p* < 0.001). Two versions of Equation P_3_ were established: P_3_ (w/o) (*χ*^2^ = 148.499, *p* < 0.001) and P_3_ (w) (*χ*^2^ = 98.663, *p* < 0.001).

### 3.4 Verification of equations P_1_, P_2_, and P_3_

The validation results showed that among the three Equation P_1_ variants, P_1_(c) demonstrated the highest SEN (88.3%), SPE (68.0%), ACC (83.1%), PPV (89.2%), and NPV (66.1%), while maintaining comparable ROC-AUC (0.830 vs. 0.842/0.842 in P_1_(w/o)/P_1_(w), all *p* < 0.001). The reduced variable count (from 10 to 6) enhanced clinical utility. In the final selected Equation P_1_, significant predictors included markedly hypoechoic feature (OR: 10.286, 95% CI: 6.118–17.296), hypoechoic feature (OR: 4.703, 95% CI: 3.190–6.932), irregular/extra-thyroidal extension (OR: 1.705, 95% CI: 1.180–2.463), enhanced posterior features (OR: 1.853, 95% CI: 1.265–2.715), and shadowing (OR: 2.809, 95% CI: 1.220–5.031), whereas lobulated shape showed nonsignificant association (OR: 1.122, 95% CI: 0.636–1.980). Isoechoic/hyperechoic pattern, oval-to-round shape, and absent posterior features were identified as independent protective factors for benign nodules.

Among the 498 non-Group 1 cases predicted by Equation P_1_, Equation P_2_ (w) demonstrated higher SEN (80.6% vs. 74.4%) and NPV (74.0% vs. 73.3%) compared to P_2_ (w/o), with comparable ROC-AUC (0.735 vs. 0.759, both *p* = 0.000). Thus, P_2_ (w) was selected to reduce missed diagnoses of malignancy. Key risk factors in Equation P_2_ included isthmus location (OR: 4.000, 95% CI: 1.475–10.843), size > 5 mm (highest risk at 5–10 mm; OR: 3.058, 95% CI: 1.671–5.596), markedly hypoechoic/hypoechoic features (OR: 20.203, 95% CI: 5.203–81.179), taller-than-wide shape (OR: 5.165, 95% CI: 2.889–9.235), microcalcifications/complex calcifications (OR: 1.199, 95% CI: 0.626–2.296), and elevated TRAb (OR: 1.628, 95% CI: 1.119–2.368). These were independent predictors of malignant nodules.

**Figure 2:**
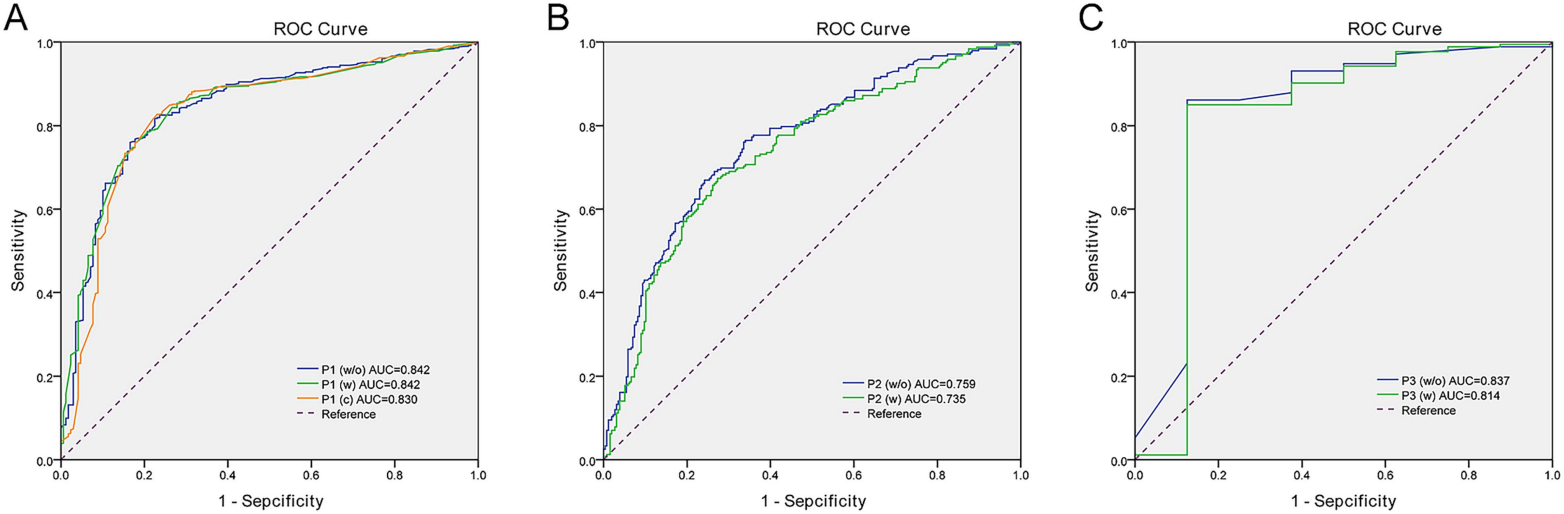
Receiver operating characteristic (ROC) curve analysis for three regression equations. **(A)** ROC curves comparing three designs (Equation P_1_) predicting Group 1 (BSRTC II). AUC values: P_1_(w/o): 0.842 (95% confidence interval [CI] 0.807–0.876), P_1_(W): 0.842 (95% CI 0.808–0.876), P_1_(C): 0.830 (95% CI 0.792–0.868). **(B)** ROC curves comparing two designs (Equation P_2_) predicting Group 4 (BSRTC V/VI). AUC values: P_2_(w/o): 0.759 (95% CI 0.717–0.801), P_2_(w): 0.735 (95% CI 0.691–0.779). **(C)** ROC curves comparing two designs (Equation P_3_) distinguishing Groups 2 (BSRTC I/III) and 3 (BSRTC IV). AUC values: P_3_(w/o): 0.837 (95% CI 0.650–1.000); P_3_(w): 0.814 (95% CI 0.599–1.000). BSRTC, Bethesda System for Reporting Thyroid Cytopathology (ROC curve plotting: SPSS 20.0, IBM; image editing: Adobe Photoshop CS5).

Among the 181 cases predicted as neither Group 1 nor 4 by Equation P_1_ and P_2_, Equation P_3_(w) showed higher SPE (96.0% vs. 95.4%) than P_3_(w/o) with similar SEN (both 37.5%), ACC (93.4% vs. 92.7%), PPV (both 2.9%), NPV (70.0% vs. 72.7%), and ROC-AUC (0.814 vs. 0.837, both *p* = 0.000). The predictive performance of Equations P_1_, P_2_ and P_3_ in the validation dataset are presented in Table 3 and Figure 2.

**Table 3:** Predictive efficacy of equations P_1_ (w/o), P_1_ (w), P_1_ (c), P_2_ (w/o), P_2_ (w), P_3_ (w/o), and P_3_ (w) in the validation dataset.

Equations	SEN (%)	SPE (%)	ACC (%)	PPV (%)	NPV (%)	ROC-AUC (95% CI)	*P*_AUC_
P_1_ (w/o)	86.5	65.7	81.3	88.2	62.0	0.842 (0.807, 0.876)	0.000
P_1_ (w)	87.3	64.5	81.5	88.0	63.0	0.842 (0.808, 0.876)	0.000
P_1_ (c)	88.3	68.0	83.1	89.2	66.1	0.830 (0.792, 0.868)	0.000
P_2_ (w/o)	74.4	66.4	70.3	67.7	73.3	0.759 (0.717, 0.801)	0.000
P_2_ (w)	80.6	52.3	66.1	61.5	74.0	0.735 (0.691, 0.779)	0.000
P_3_ (w/o)	37.5	95.4	92.8	2.9	72.7	0.837 (0.650, 1.000)	0.000
P_3_ (w)	37.5	96.0	93.4	2.9	70.0	0.814 (0.599, 1.000)	0.000

ACC, accuracy; NPV, negative predictive value; P_AUC_, *p*-value for area under the curve; PPV, positive predictive value; ROC-AUC, receiver operating characteristic-area under the curve; SEN, sensitivity; SPE, specificity.

### 3.5 Overall efficacy of the B-Model

For the validation dataset, the number of cases correctly predicted by the B-Model were 115, 91, 3, and 195 in Groups 1, 2, 3, and 4, respectively. The prediction results of B-Model in the validation dataset are presented in Table 4. True Group 2/3 cases were 255, and true Group 2/3 cases incorrectly classified as Group 1/4 by B-Model was 153. The rFNA reduction rate was 40%.

**Table 4:** The prediction results of B-Model in the validation dataset.

Prediction grouping	Actual grouping			
	Group 1	Group 2	Group 3	Group 4
Group 1	115	50	3	6
Group 2	30	91	5	45
Group 3	2	3	3	2
Group 4	22	95	5	195

## 4 Discussion

US remains the primary imaging tool for TN risk stratification. While certain US features are associated with malignancy, most nodules still require FNA for definitive diagnosis. This study bridges this gap by integrating clinical, biochemical, and US features into a cascaded multivariable logistic regression model (B-Model) for pre-FNA prediction of BSRTC categories.

Operationally, the B-Model links three logistic regression equations in sequence. At the point of use, clinicians input the available clinical, ultrasound, and biochemical variables; the model sequentially evaluates benign vs. non-benign (Equation P_1_), malignant vs. non-malignant (Equation P_2_), and follicular neoplasm vs. indeterminate/atypia (Equation P_3_). A fixed threshold of 0.5 is applied at each step, ensuring that every nodule is ultimately assigned to one, and only one, predicted BSRTC group.

**Figure 3:**
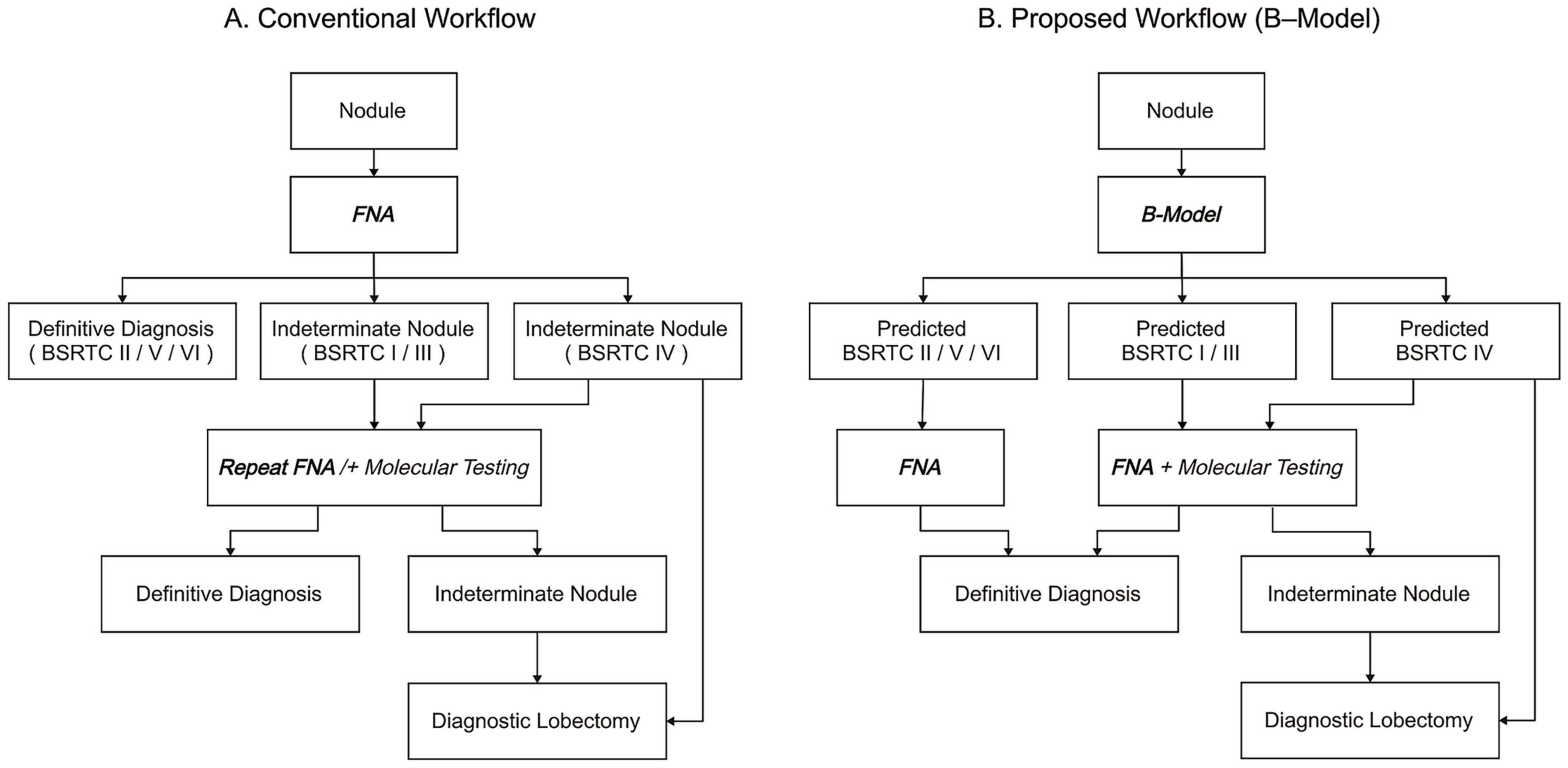
Diagnostic workflows for thyroid nodular diseases. **(A)** Conventional workflow based on fine-needle aspiration (FNA). Indeterminate results (BSRTC I, III, IV) require repeat FNA/and molecular testing, with unresolved nodules often proceeding to diagnostic lobectomy. **(B)** Proposed workflow using B-Model. Nodules are stratified into predicted BSRTC II/V/VI (direct FNA), BSRTC I/III (FNA + molecular testing), and BSRTC IV (molecular testing or direct diagnostic lobectomy), providing a more streamlined and individualized management strategy. Notably, in the B-Model, each nodule theoretically requires only a single FNA, avoiding repeated punctures. FNA, fine-needle aspiration; BSRTC, Bethesda System for Reporting Thyroid Cytopathology [Flowchart design: Boardmix Online Platform (https://boardmix.cn)].

As illustrated in Figure 3 this structured, pre-FNA assignment provides direct guidance for patient management. In contrasts to the conventional workflow, where indeterminate cytology (BSRTC I, III, IV) often necessitate rFNA and may ultimately proceed to diagnostic lobectomy, the B-Model enables early identification of nodules likely to yield indeterminate results. Such cases can be directly triaged to FNA plus molecular testing or diagnostic lobectomy, thereby avoiding redundant punctures. In the validation dataset, this approach reduced the rFNA by 40.0%, minimizing patient trauma and conserving healthcare resources. Importantly, the B-Model theoretically requires only a single FNA per nodule, representing a significant advancement in clinical efficiency.

A key methodological consideration was the reduction of cumulative errors inherent to cascaded regression. To mitigate this risk, BSRTC categories with similar clinical management strategies were merged (BSRTC I with III, and BSRTC V with VI), reducing six categories to four groups (1, 8, 11, 18). This consolidation balanced statistical robustness clinical practicality and minimized propagation error. Similar sequential or multi-step logistic regression strategies have been applied successfully in other medical domains, supporting both interpretability and transparency of the modeling process (19–21).

Although machine learning and deep learning methods such as convolutional neural networks (CNNs) have been increasingly applied in radiomics, they remain limited by several drawbacks (22–26). First, the ‘black-box’ nature of CNNs prevents transparent identification of the imaging features driving classification, thereby reducing interpretability. Second, overfitting may arise when models are over-parameterized, which undermines generalizability (26–29). In contrast, we selected a cascaded logistic regression model because it provides transparent and interpretable results that facilitate the training of junior clinicians; its sequential structure mimics a decision tree, which helps handle data imbalance while preserving a linear framework; and it also offers a necessary foundation for subsequent AI research, enabling insight into the underlying decision logic before moving toward more advanced algorithms (17, 20, 30).

Beyond diagnostic utility, the B-Model highlighted certain features that deserve further clinical attention. Equation P_2_ identified younger age, isthmus location, and small nodule size (particularly 5–10 mm) as predictors of malignancy. While some study have reported similar findings, one possible explanation for this observation in our cohort is the relatively high proportion of sub-centimeter and isthmus-located nodules (31–34). This indicated that conventional size–risk associations, which are largely derived from nodules ≥1 cm, may not fully capture the risk pattern of microcarcinomas. As a result, the diagnosis of microcarcinomas remains challenging, particularly for junior clinicians (35). By incorporating these features, our model provides intuitive “rules of thumb” that support structured image interpretation and enhance diagnostic confidence, especially for nodules ≤1 cm. Thus, the B-Model serves not only as a decision-support system but also as a valuable teaching aid.

This study has limitations. First, although the training and validation cohorts were largely comparable, differences were observed in FT4, A-TG, and A-TPO levels. These variations likely reflect case-mix shifts from time-based cohort division and assay-related variability in laboratory testing, but they were confined to biochemical indicators and did not affect model performance. Second, as a single-center study, variability in ultrasonography and pathologic interpretation may limit generalizability. Third, collinearity and potential confounding were not explicitly tested, though variables were selected based on clinical relevance and univariable screening, and regression coefficients remained stable. Finally, while the B-Model reduced rFNA by 40% under retrospective conditions, its real-world effectiveness and operational feasibility requires validation through prospective multicenter studies.

In conclusion, we developed a cascaded logistic regression model and demonstrated its effectiveness. By integrating clinical, ultrasound, and biochemical indicators, the B-Model enabled pre-FNA prediction of BSRTC categories, thereby optimizing the diagnostic workflow for TNs, reducing unnecessary FNAs, and advancing precision medicine in TN management.

## Data Availability

The raw data supporting the conclusions of this article will be made available by the authors, without undue reservation.
