# Supplementary material for: A cascaded clinical-ultrasound-biochemical model for precise prediction before thyroid nodule fine-needle aspiration biopsy
Source: Front Med (Lausanne). 2025 Sep 18;12:1641266. doi: 10.3389/fmed.2025.1641266 (PMC12488719; doi:10.3389/fmed.2025.1641266)
Supplement: Supplementary file 1 [file Table_1.docx]

Supplementary Material

# Supplementary Tables

**Supplementary Table 1.** Pairwise comparison of the differences in the echotexture of the thyroid, FT4, and A-TG across different groups in the training dataset

| Groups | *P-*value | | |
| --- | --- | --- | --- |
|  | Echotexture of the Thyroid | FT4 | ATG |
| 1 and 2 | 0.882 | 0.224 | 0.169 |
| 1 and 3 | 0.387 | 0.930 | 0.521 |
| 1 and 4 | 0.047* | 0.413 | 0.046* |
| 2 and 3 | 0.390 | 0.528 | 0.788 |
| 2 and 4 | 0.063 | 0.032* | 0.654 |
| 3 and 4 | 1.000 | 0.794 | 0.753 |

* *P*-value < 0.05

FT4, free thyroxine; A-TG, thyroglobulin antibody.**Supplementary Table 2.** Scoring for Categorical Variables

|  | Score |
| --- | --- |
| Sex | Female = 0*; Male = 1 |
| Echotexture of Thyroid | Homogeneous = 0*; Heterogeneous = 1 |
| Lobe | Right = 0*; Left =1; Isthmus = 2 |
| Position | Inferior = 0*; Superior = 1; Middle = 2 |
| Distance from the capsule | >2 mm = 0*; ≤2 mm =1 |
| Maximum diameter (mm) | ≤5.0 = 0*; 5.1–10.0 = 1; 10.1–40.0 = 2; >40.0 = 3 |
| Composition | Solid = 0*; Predominantly solid =1; Predominantly cystic = 2; Spongiform = 3 |
| Echogenicity | Isoechoic/hyperechoic = 0*; Hypoechoic = 1; Markedly hypoechoic = 2 |
| Echotexture of nodule | Homogeneous = 0*; Heterogeneous = 1 |
| Margin | Smooth = 0*; Ill-defined = 1 |
| Shape | Oval-to-round = 0*; Lobulated = 1; Irregular/extra-thyroidal extension = 2 |
| Orientation | Wider-than-tall = 0*; Taller-than-wide = 1 |
| Calcifications | Absent = 0*; Macrocalcifications = 1; Microcalcifications = 2; Peripheral calcifications = 3; More than two forms = 4 |
| Posterior features | Absent = 0*; Enhancement = 1; Shadowing =2 |
| Halo | Absent = 0*; Uniform halo = 1; Uneven halo = 2 |
| Adler grading | Grade 0 = 0*; Grade 1 = 2; Grade 2 = 3; Grade 3 = 4 |

* Based on clinical significance or published reports, the indicated independent variable is considered as baseline risk reference value and scored as 0.

**Supplementary Table 3**. Coefficient of risk factor in the multivariate binary logistic regression equation for P_1_ (w/o)

|  | β coefficient | *P-*value | OR | 95% CI | |
| --- | --- | --- | --- | --- | --- |
|  |  |  |  | Upper limit | Lower limit |
| Echotexture of thyroid | −0.487 | 0.002 | 0.615 | 0.453 | 0.835 |
| Position | ·· | 0.037 | ·· | ·· | ·· |
| Superior | 0.492 | 0.012 | 1.635 | 1.113 | 2.403 |
| Middle | 0.082 | 0.580 | 1.086 | 0.811 | 1.452 |
| Distance from the capsule | 0.353 | 0.021 | 1.423 | 1.054 | 1.921 |
| Size (mm) | ·· | 0.000 | ·· | ·· | ·· |
| 5.1–10.0 | −0.736 | 0.001 | 0.479 | 0.307 | 0.747 |
| 10.1–40.0 | −1.231 | 0.000 | 0.292 | 0.169 | 0.505 |
| >40.0 | −1.038 | 0.006 | 0.354 | 0.169 | 0.744 |
| Echogenicity | ·· | 0.000 | ·· | ·· | ·· |
| Markedly hypoechoic | 2.238 | 0.000 | 9.371 | 5.561 | 15.791 |
| Hypoechoic | 1.615 | 0.000 | 5.026 | 3.446 | 7.330 |
| Echotexture of nodule | −0.419 | 0.003 | 0.658 | 0.499 | 0.868 |
| Orientation | 0.789 | 0.000 | 2.202 | 1.591 | 3.047 |
| Calcifications | ·· | 0.047 | ·· | ·· | ·· |
| Macrocalcifications | −0.042 | 0.867 | 0.959 | 0.588 | 1.564 |
| Microcalcifications | 0.655 | 0.004 | 1.925 | 1.239 | 2.992 |
| Peripheral calcifications | −0.479 | 0.457 | 0.619 | 0.175 | 2.188 |
| More than two forms | 19.685 | 0.998 | 354,147  670.210 | 0.000 | 0.000 |
| Posterior features | ·· | 0.001 | ·· | ·· | ·· |
| Enhancement | 0.485 | 0.010 | 1.624 | 1.124 | 2.348 |
| Shadowing | 1.276 | 0.004 | 3.582 | 1.520 | 8.440 |
| Adler grading | ·· | 0.011 | ·· | ·· | ·· |
| Grade 1 | 0.094 | 0.600 | 1.098 | 0.774 | 1.559 |
| Grade 2 | 0.371 | 0.095 | 1.450 | 0.937 | 2.242 |
| Grade 3 | 0.783 | 0.002 | 2.189 | 1.344 | 3.563 |
| Constant | −0.247 | 0.412 | 0.781 | ·· | ·· |

* *P-*value < 0.05, indicating a variable with a significant impact on grouping, which is included as an independent variable in the logistic regression equation.

CI, confidence interval; OR, odds ratio.

**Supplementary Table 4.** Coefficient of risk factors in the multivariate binary logistic regression equation for P_1_ (w)

|  | β coefficient | *P-*value | OR | 95% CI | |
| --- | --- | --- | --- | --- | --- |
|  |  |  |  | Upper limit | Lower limit |
| Echotexture of thyroid | −0.712 | 0.017 | 0.490 | 0.274 | 0.878 |
| Size (mm) | ·· | 0.000 | ·· | ·· | ·· |
| 5.1–10.0 | −0.296 | 0.513 | 0.744 | 0.306 | 1.805 |
| 10.1–40.0 | −1.549 | 0.000 | 0.212 | 0.090 | 0.500 |
| >40.0 | −1.213 | 0.030 | 0.297 | 0.099 | 0.892 |
| Composition | ·· | 0.052 | ·· | ·· | ·· |
| Predominantly solid | −0.436 | 0.181 | 0.647 | 0.342 | 1.224 |
| Predominantly cystic | −0.224 | 0.606 | 0.799 | 0.341 | 1.874 |
| Spongiform | −1.286 | 0.007 | 0.276 | 0.109 | 0.700 |
| Echogenicity | ·· | 0.000 | ·· | ·· | ·· |
| Markedly hypoechoic | 3.174 | 0.000 | 23.910 | 7.492 | 76.305 |
| Hypoechoic | 1.652 | 0.000 | 5.216 | 2.882 | 9.440 |
| Shape | ·· | 0.013 | ·· | ·· | ·· |
| Lobulated | −0.237 | 0.621 | 0.789 | 0.309 | 2.017 |
| Irregular/extra-thyroidal extension | 1.578 | 0.004 | 4.844 | 1.642 | 14.290 |
| Posterior features | ·· | 0.000 | ·· | ·· | ·· |
| Enhancement | 1.008 | 0.000 | 2.741 | 1.554 | 4.834 |
| Shadowing | 2.021 | 0.018 | 7.548 | 1.418 | 40.175 |
| Constant | 0.586 | 0.203 | 1.798 | ·· | ·· |

* *P-*value < 0.05, indicating a variable with a significant impact on grouping, which is included as an independent variable in the logistic regression equation.

CI, confidence interval; OR, odds ratio.

**Supplementary Table 5.** Coefficient of risk factors in the multivariate binary logistic regression equation for P_1_ (c)

|  | β coefficient | *P-*value | OR | 95% CI | |
| --- | --- | --- | --- | --- | --- |
|  |  |  |  | Upper limit | Lower limit |
| Echotexture of thyroid | −0.548 | 0.000 | 0.578 | 0.428 | 0.781 |
| Size (mm) | ·· | 0.000 | ·· | ·· | ·· |
| 5.1–10.0 | −0.828 | 0.000 | 0.437 | 0.287 | 0.664 |
| 10.1–40.0 | −1.222 | 0.000 | 0.295 | 0.187 | 0.465 |
| >40.0 | −0.928 | 0.007 | 0.395 | 0.201 | 0.777 |
| Composition | ·· | 0.097 | ·· | ·· | ·· |
| Predominantly solid | −0.466 | 0.042 | 0.627 | 0.400 | 0.984 |
| Predominantly cystic | −0.320 | 0.340 | 0.726 | 0.377 | 1.400 |
| Spongiform | −0.649 | 0.042 | 0.523 | 0.279 | 0.978 |
| Echogenicity | ·· | 0.000 | ·· | ·· | ·· |
| Markedly hypoechoic | 2.331 | 0.000 | 10.286 | 6.118 | 17.296 |
| Hypoechoic | 1.548 | 0.000 | 4.703 | 3.190 | 6.932 |
| Shape | ·· | 0.018 | ·· | ·· | ·· |
| Lobulated | 0.115 | 0.691 | 1.122 | 0.636 | 1.980 |
| Irregular/extra-thyroidal extension | 0.533 | 0.004 | 1.705 | 1.180 | 2.463 |
| Posterior features | ·· | 0.000 | ·· | ·· | ·· |
| Enhancement | 0.617 | 0.002 | 1.853 | 1.265 | 2.715 |
| Shadowing | 1.033 | 0.007 | 2.809 | 1.330 | 5.931 |
| Constant | 0.517 | 0.052 | 1.677 | ·· | ·· |

* *P-*value < 0.05, indicating a variable with a significant impact on grouping, which is included as an independent variable in the logistic regression equation.

CI, confidence interval; OR, odds ratio.

**Supplementary Table 6.** Coefficient of risk factors in the multivariate binary logistic regression equation for P_2_ (w/o)

|  | β coefficient | P-value | OR | 95% CI | |
| --- | --- | --- | --- | --- | --- |
|  |  |  |  | Upper limit | Lower limit |
| Age | −0.031 | 0.000 | 0.970 | 0.958 | 0.981 |
| Echotexture of thyroid | −0.479 | 0.004 | 0.620 | 0.446 | 0.860 |
| Lobe | ·· | 0.000 | ·· | ·· | ·· |
| Left | −0.183 | 0.210 | 0.833 | 0.626 | 1.108 |
| Isthmus | 1.183 | 0.000 | 3.263 | 1.849 | 5.756 |
| Size (mm) | ·· | 0.000 | ·· | ·· | ·· |
| 5.1–10.0 | 0.754 | 0.000 | 2.125 | 1.512 | 2.987 |
| 10.1–40.0 | 0.805 | 0.002 | 2.237 | 1.329 | 3.766 |
| >40.0 | 0.492 | 0.503 | 1.635 | 0.387 | 6.906 |
| Echogenicity | ·· | 0.000 | ·· | ·· | ·· |
| Markedly hypoechoic | 2.717 | 0.000 | 15.142 | 5.848 | 39.205 |
| Hypoechoic | 2.490 | 0.000 | 12.059 | 4.804 | 30.269 |
| Shape | ·· | 0.069 | ·· | ·· | ·· |
| Lobulated | 0.638 | 0.079 | 1.892 | 0.928 | 3.859 |
| Irregular/extra-thyroidal extension | 0.275 | 0.085 | 1.317 | 0.962 | 1.802 |
| Orientation | 1.045 | 0.000 | 2.844 | 2.066 | 3.916 |
| Calcifications | ·· | 0.000 | ·· | ·· | ·· |
| Macrocalcifications | −0.450 | 0.055 | 0.638 | 0.403 | 1.009 |
| Microcalcifications | 0.570 | 0.002 | 1.769 | 1.233 | 2.538 |
| Peripheral calcifications | −1.606 | 0.049 | 0.201 | 0.041 | 0.990 |
| More than two forms | 1.386 | 0.019 | 4.001 | 1.261 | 12.691 |
| Adler grading | ·· | 0.000 | ·· | ·· | ·· |
| Grade 1 | 0.210 | 0.270 | 1.233 | 0.850 | 1.790 |
| Grade 2 | −0.466 | 0.100 | 0.628 | 0.361 | 1.093 |
| Grade 3 | −1.343 | 0.000 | 0.261 | 0.126 | 0.540 |
| Constant | −2.123 | 0.000 | 0.120 | ·· | ·· |

* *P-*value < 0.05, indicating a variable with a significant impact on grouping, which is included as an independent variable in the logistic regression equation.

CI, confidence interval; OR, odds ratio.

**Supplementary Table 7.** Coefficient of risk factor in the multivariate binary logistic regression equation for P_2_ (w)

|  | β coefficient | *P-*value | OR | 95% CI | |
| --- | --- | --- | --- | --- | --- |
|  |  |  |  | Upper limit | Lower limit |
| Age | −0.033 | 0.003 | 0.968 | 0.947 | 0.989 |
| Echotexture of thyroid | −0.571 | 0.061 | 0.565 | 0.311 | 1.027 |
| Lobe | ·· | 0.006 | ·· | ·· | ·· |
| Left | −0.273 | 0.292 | 0.761 | 0.458 | 1.265 |
| Isthmus | 1.386 | 0.006 | 4.000 | 1.475 | 10.843 |
| Size (mm) | ·· | 0.004 | ·· | ·· | ·· |
| 5.1–10.0 | 1.118 | 0.000 | 3.058 | 1.671 | 5.596 |
| 10.1–40.0 | 0.869 | 0.021 | 2.385 | 1.137 | 5.004 |
| >40.0 | 0.714 | 0.530 | 2.042 | 0.220 | 18.927 |
| Echogenicity | ·· | 0.000 | ·· | ·· | ·· |
| Markedly hypoechoic | 3.006 | 0.000 | 20.203 | 5.028 | 81.179 |
| Hypoechoic | 2.754 | 0.000 | 15.699 | 4.256 | 57.910 |
| Orientation | 1.642 | 0.000 | 5.165 | 2.889 | 9.235 |
| Calcifications | ·· | 0.060 | ·· | ·· | ·· |
| Macrocalcifications | −1.020 | 0.032 | 0.361 | 0.142 | 0.914 |
| Microcalcifications | 0.181 | 0.585 | 1.199 | 0.626 | 2.296 |
| Peripheral calcifications | −2.416 | 0.060 | 0.089 | 0.007 | 1.108 |
| More than two forms | 0.352 | 0.716 | 1.422 | 0.213 | 9.476 |
| TSH | −0.192 | 0.003 | 0.825 | 0.726 | 0.938 |
| FT4 | −0.124 | 0.012 | 0.884 | 0.803 | 0.973 |
| TG | −0.005 | 0.002 | 0.995 | 0.992 | 0.998 |
| TRAb | 0.487 | 0.011 | 1.628 | 1.119 | 2.368 |
| Constant | −0.141 | 0.911 | 0.868 | ·· | ·· |

* *P-*value < 0.05, indicating a variable with a significant impact on grouping, which is included as an independent variable in the logistic regression equation.

CI, confidence interval; FT4, free thyroxine; OR, odds ratio; TG, thyroglobulin; TRAb, thyrotropin receptor antibody; TSH, thyroid-stimulating hormone.

**Supplementary Table 8.** Coefficient of risk factors in the multivariate binary logistic regression equation for P_3_ (w/o)

|  | β coefficient | *P-*value | OR | 95% CI | |
| --- | --- | --- | --- | --- | --- |
|  |  |  |  | Upper limit | Lower limit |
| Sex | −0.770 | 0.031 | 0.463 | 0.230 | 0.934 |
| Size (mm) | ·· | 0.176 | ·· | ·· | ·· |
| 5.1–10.0 | −1.976 | 0.065 | 0.139 | 0.017 | 1.133 |
| 10.1–40.0 | −1.996 | 0.071 | 0.136 | 0.016 | 1.188 |
| Lobe | −2.635 | 0.030 | 0.072 | 0.007 | 0.775 |
| Echogenicity | ·· | 0.000 | ·· | ·· | ·· |
| Markedly hypoechoic | −2.223 | 0.000 | 0.108 | 0.035 | 0.331 |
| Hypoechoic | −1.754 | 0.000 | 0.173 | 0.075 | 0.400 |
| Posterior features | ·· | 0.002 | ·· | ·· | ·· |
| Enhancement | −1.203 | 0.001 | 0.300 | 0.151 | 0.597 |
| Shadowing | 0.100 | 0.900 | 1.105 | 0.231 | 5.277 |
| Adler grading | ·· | 0.000 | ·· | ·· | ·· |
| Grade 1 | −1.757 | 0.002 | 0.173 | 0.056 | 0.530 |
| Grade 2 | −2.696 | 0.000 | 0.067 | 0.021 | 0.218 |
| Grade 3 | −3.065 | 0.000 | 0.047 | 0.014 | 0.160 |
| Constant | 7.555 | 0.000 | 1909.431 | ·· | ·· |

* *P-*value < 0.05, indicating a variable with a significant impact on grouping, which is included as an independent variable in the logistic regression equation.

CI, confidence interval; OR, odds ratio.

**Supplementary Table 9.** Coefficient of risk factors in the multivariate binary logistic regression equation for P_3_ (w)

|  | β coefficient | *P-*value | OR | 95% CI | |
| --- | --- | --- | --- | --- | --- |
|  |  |  |  | Upper limit | Lower limit |
| Sex | −1.401 | 0.029 | 0.246 | 0.070 | 0.863 |
| Echogenicity | ·· | 0.001 | ·· | ·· | ·· |
| Markedly hypoechoic | −2.433 | 0.008 | 0.088 | 0.014 | 0.532 |
| Hypoechoic | −2.204 | 0.000 | 0.110 | 0.032 | 0.375 |
| Posterior features | ·· | 0.008 | ·· | ·· | ·· |
| Enhancement | −1.664 | 0.002 | 0.189 | 0.066 | 0.540 |
| Shadowing | −0.715 | 0.569 | 0.489 | 0.042 | 5.731 |
| Adler grading | ·· | 0.000 | ·· | ·· | ·· |
| Grade 1 | −1.515 | 0.180 | 0.220 | 0.024 | 2.009 |
| Grade 2 | −3.628 | 0.000 | 0.027 | 0.004 | 0.165 |
| Grade 3 | −3.899 | 0.000 | 0.020 | 0.003 | 0.143 |
| FT3 | −0.421 | 0.024 | 0.656 | 0.455 | 0.947 |
| A-TPO | −0.003 | 0.025 | 0.997 | 0.995 | 1.000 |
| Constant | 9.354 | 0.000 | 11,541.682 | ·· | ·· |

* *P-*value < 0.05, indicating a variable with a significant impact on grouping, which is included as an independent variable in the logistic regression equation.

A-TPO, antithyroid peroxidase autoantibody; CI, confidence interval; FT3, free triiodothyronine; OR, odds ratio.

**Supplementary Table 10.** Prediction results of P_1_ (w/o), P_1_ (w) and P_1_ (c) in the validation dataset

| Prediction grouping | | Actual grouping | |
| --- | --- | --- | --- |
|  |  | Not Group 1 (n = 503) | Group 1 (n = 169) |
| P_1_ (w/o) | Not Group 1 (n = 493) | 435 | 58 |
|  | Group 1 (n = 179) | 68 | 111 |
| P_1_ (w) | Not Group 1 (n = 499) | 439 | 60 |
|  | Group 1 (n = 173) | 64 | 109 |
| P_1_ (c) | Not Group 1 (n = 498) | 444 | 54 |
|  | Group 1 (n = 174) | 59 | 115 |

**Supplementary Table 11.** Prediction results of P_2_ (w/o) and P_2_ (w) in the validation dataset

| Prediction grouping | | Actual grouping | |
| --- | --- | --- | --- |
|  |  | Group 4 (n = 242) | Not Group 1 or 4 (n = 256) |
| P_2_ (w/o) | Group 4 (n = 266) | 180 | 86 |
|  | Not Group 1 or 4 (n = 232) | 62 | 170 |
| P_2_ (w) | Group 4 (n = 317) | 195 | 122 |
|  | Not Group 1 or 4 (n = 181) | 47 | 134 |

**Supplementary Table 12.** Prediction results of P_3_ (w/o) and P_3_ (w) in the validation dataset

| Prediction grouping | | Actual grouping | |
| --- | --- | --- | --- |
|  |  | Group 3 (n = 8) | Group 2 (n = 173) |
| P_3_ (w/o) | Group 2 (n = 170) | 5 | 165 |
|  | Group 3 (n = 11) | 3 | 8 |
| P_3_ (w) | Group 2 (n = 171) | 5 | 166 |
|  | Group 3 (n = 10) | 3 | 7 |
