# Supplementary material for: A cascaded clinical-ultrasound-biochemical model for precise prediction before thyroid nodule fine-needle aspiration biopsy
Source: Front Med (Lausanne). 2025 Sep 18;12:1641266. doi: 10.3389/fmed.2025.1641266 (PMC12488719; doi:10.3389/fmed.2025.1641266)
Supplement: Supplementary file 2 [file Table_2.docx]

Supplementary Material

# Supplementary File – Logistic Regression Equations of the B-Model

The three logistic regression equations constituting the B-Model are presented below. Each equation corresponds to one stage of the cascaded framework:

## Equation P_1_: Distinguishes benign from non-benign nodules

y_1_ = ＋ 0.517 － 0.548 × (Heterogeneous of Thyroid)

－ 0.828 × (Size = (5.1mm – 10.0mm)) / － 1.222 × (Size = (10.1mm – 40.0mm)) / － 0.928 × (Size = ( > 40.0mm))

－ 0.466 × (Predominantly Solid) / － 0.320 × (Predominantly Cystic) / － 0.649 × (Spongiform)

＋ 2.331 × (Markedly Hypoechoic) / ＋ 1.548 × (Hypoechoic)

＋ 0.115 × (Lobulated) / ＋ 0.533 × (Irregular/ Extra-thyroidal Extension)

＋ 0.617 × (Echo Enhancement Posterior to the Nodule) / ＋1.033 × (Shadow Posterior to the Nodule)

P_1_ =$\text{ }\frac{\text{e}^{\text{y}_{\text{1}}}}{\text{1+}\text{e}^{\text{y}_{\text{1}}}\text{ }}$

## Equation P_2_: Differentiates malignant from non-malignant nodules

y_2_ = － 0.141 － 0.033 × (Age)

－ 0.571 × (Heterogeneous of Thyroid)

－ 0.273 × (Located in Left Lobe) / ＋ 1.386 × (Located in Isthmus)

＋ 1.118 × (Size = (5.1mm – 10.0mm)) / ＋ 0.869 × (Size = (10.1mm – 40.0mm)) / ＋ 0.714 × (Size = ( > 40.0mm))

＋ 3.006 × (Markedly Hypoechoic) / ＋2.754 × (Hypoechoic)

＋ 1.642 × (Taller-than-wide)

＋ 1.020 × (Macrocalcifications) / ＋ 0.181 × (Microcalcifications) / － 2.416 × (Peripheral Calcifications) / ＋ 0.352 × (More Than Two Forms of Calcifications)

－ 0.192 × (TSH)

－ 0.124 × (FT4)

－ 0.005 × (TG)

＋ 0.487 × (TRAB)

P_2_ =$\text{ }\frac{\text{e}^{\text{y}_{\text{2}}}}{\text{1+}\text{e}^{\text{y}_{\text{2}}}\text{ }}$

## Equation P_3_: Separates follicular neoplasm from indeterminate/atypia nodules

y_3_ = ＋ 9.354 - 1.401 × (Sex)

－ 2.433 × (Markedly Hypoechoic) / － 2.204 × (Hypoechoic)

－ 1.664 × (Echo Enhancement Posterior to the Nodule) / － 0.715 × (Shadow Posterior to the Nodule)

－ 1.515 × (Adler Grade 1) / － 3.628 × (Adler Grade 2) / － 3.899 × (Adler Grade 3)

－ 0.421 × (FT3)

－ 0.003 × (A-TPO)

P_3_ =$\text{ }\frac{\text{e}^{\text{y}_{\text{3}}}}{\text{1+}\text{e}^{\text{y}_{\text{3}}}\text{ }}$
